# Combination of dasatinib and quercetin improves cognitive abilities in aged male Wistar rats, alleviates inflammation and changes hippocampal synaptic plasticity and histone H3 methylation profile

**DOI:** 10.18632/aging.203835

**Published:** 2022-01-18

**Authors:** Adam Krzystyniak, Malgorzata Wesierska, Gregory Petrazzo, Agnieszka Gadecka, Magdalena Dudkowska, Anna Bielak-Zmijewska, Grazyna Mosieniak, Izabela Figiel, Jakub Wlodarczyk, Ewa Sikora

**Affiliations:** 1 Laboratory of Molecular Bases of Aging, Nencki Institute of Experimental Biology, Polish Academy of Sciences, Warsaw 02-093, Poland; 2 Laboratory of Neuropsychology, Nencki Institute of Experimental Biology, Polish Academy of Sciences, Warsaw 02-093, Poland; 3 Laboratory of Cell Biophysics, Nencki Institute of Experimental Biology, Polish Academy of Sciences, Warsaw 02-093, Poland

**Keywords:** aging, brain, plasticity, cognition, memory, hippocampus, SASP, senescence

## Abstract

Aging is associated with cognitive decline and accumulation of senescent cells in various tissues and organs. Senolytic agents such as dasatinib and quercetin (D+Q) in combination have been shown to target senescent cells and ameliorate symptoms of aging-related disorders in mouse models. However, the mechanisms by which senolytics improve cognitive impairments have not been fully elucidated particularly in species other than mice. To study the effect of senolytics on aging-related multifactorial cognitive dysfunctions we tested the spatial memory of male Wistar rats in an active allothetic place avoidance task. Here we report that 8 weeks treatment with D+Q alleviated learning deficits and memory impairment observed in aged animals. Furthermore, treatment with D+Q resulted in a reduction of the peripheral inflammation measured by the levels of serum inflammatory mediators (including members of senescent cell secretome) in aged rats. Significant improvements in cognitive abilities observed in aged rats upon treatment with D+Q were associated with changes in the dendritic spine morphology of the apical dendritic tree from the hippocampal CA1 neurons and changes in the level of histone H3 trimethylation at lysine 9 and 27 in the hippocampus. The beneficial effects of D+Q on learning and memory in aged rats were long-lasting and persisted at least 5 weeks after the cessation of the drugs administration. Our results expand and provide new insights to the existing knowledge associated with effects of senolytics on alleviating age-related associated cognitive dysfunctions.

## INTRODUCTION

Aging is the major risk factor for cancer, cardiovascular disease, diabetes, and neurodegenerative disorders associated with multiple cognitive impairments that lead to significant disabilities and lower quality of life. According to the UN estimates, the world population of people above 65 years old will double in the next 30 years and is expected to exceed 1,5 billion worldwide by 2050 (UN, 2019). However, if the prevalence of the COVID-19 infection continues to grow, this could impact life expectancy and change these estimations [1]. Aging is associated with cognitive changes that have been well described in both humans and laboratory animals. There are different aspects of cognition that gradually decline with age including, short- and long-term memory, learning, conceptual reasoning, processing speed, and psychomotor abilities [2]. Since normal aging has not been associated with significant changes in the number of brain cells [3], the accompanying cognitive impairments are believed to result from alterations in the cellular and molecular mechanisms of brain plasticity. Dendritic protrusions called dendritic spines that form synapses have distinct functional characteristics depending on their shape. The longer and thinner dendritic spines are immature and represent new, more unstable connections, whereas bigger and mushroom-like spines usually are associated with stable synapses. Both, the least mature and most mature spines are underrepresented in the aged hippocampus and frontal cortex, two brain regions critical for cognitive abilities [4, 5]. The exceptional plasticity of mature neurons is controlled by activity-dependent changes in gene expression. One of the mechanisms that allow rapid changing of the accessibility of genes to the transcriptional machinery is post-translational modification of histones such as methylation. Methylation of histones, primarily of their lysine (K) residues, regulates memory formation and synaptic plasticity [6]. In particular, methylation of histone H3 has been implicated in age-associated cognitive decline in mice. Aging causes upregulation of H3 trimethylation at lysine 3 (H3K9me3) [7] and downregulation at lysine 27 (H3K27me3) [8] in the mouse hippocampus. Recently it has been shown that the decrease in the H3K9me3 by systemic administration of an inhibitor of the principal enzyme responsible for the trimethylation of H3K9 (histone methyltransferase SUV39H1) improved memory performance in aged, but not young mice [9].

It has been postulated that the accumulation of senescent cells with age may be the main culprit of aging. Indeed, the accumulation of senescent cells was observed in healthy tissue of old animals and humans and at sites of many age-related pathologies, including the brains of animal models of Parkinson’s and Alzheimer’s diseases (reviewed in [10]). Proinflammatory cytokines such as TNF-α, IL-6 and IL-1, which are the components of so termed senescence-associated secretory phenotype - SASP when constitutively secreted by senescent cells [11], participate in a low grade inflammatory state, termed inflammaging [12]. Inflammaging may be one of the major mechanisms responsible for diseases of advanced age. Multiple studies have shown that elevated levels of inflammatory cytokines regulate learning and memory by modulating synaptic plasticity (e.g. [13, 14]).

Senolytics are synthetic or natural compounds, which are able to target senescent cells [15, 16]. Their primary mechanism of action relies on inducing apoptotic cell death by affecting several different pro-survival pathways [17]. However, it is postulated that those compounds may also exert senostatic activity, by inhibiting SASP, which otherwise leads to spreading of senescence due to the paracrine effect of senescence progression [18]. Till now several published animal studies have shown that the eradication of senescent cells alleviates many aging-related pathologies [19–21]. The natural senolytic fisetin and a combination of dasatinib and quercetin (D+Q) treatment improve cognitive ability in mouse models of Alzheimer’s disease and dementia [22, 23]. Fisetin or D+Q appear to be the most effective senolytics tested in many preclinical studies and growing body of clinical trials [20, 24]. In a recent study Ogrodnik et al. provided the first evidence for beneficial effects of D+Q in alleviating age-associated cognitive decline in mice, tested in water - escape version of the Stone T-maze [25]. In the present study, we hypothesized that D+Q treatment alleviates cognitive decline in rats by decreasing senescence-associated phenotype burden. To verify this hypothesis we have used a method that allows complex analysis of different aspects of cognition, namely spatial learning and memory, in the active allothetic place avoidance task (AAPAT). In the AAPAT test, formation of spatial representation requires a fully functional hippocampus [26]. To better understand the mechanism of action of D+Q we sought to investigate whether improved cognition following D+Q treatment in aged animals is associated with changes in the peripheral level of inflammation, brain synaptic plasticity and regulation of gene expression involved in modulation of synaptic plasticity. Our results provide new insights into the mechanisms of improvement of cognitive abilities by senolytics and offer valuable data confirming the effectiveness of D+Q treatment for age-associated learning and memory deficits in rats.

## RESULTS

### D+Q alleviates age-associated cognitive deficits in aged rats

**Figure 1:**
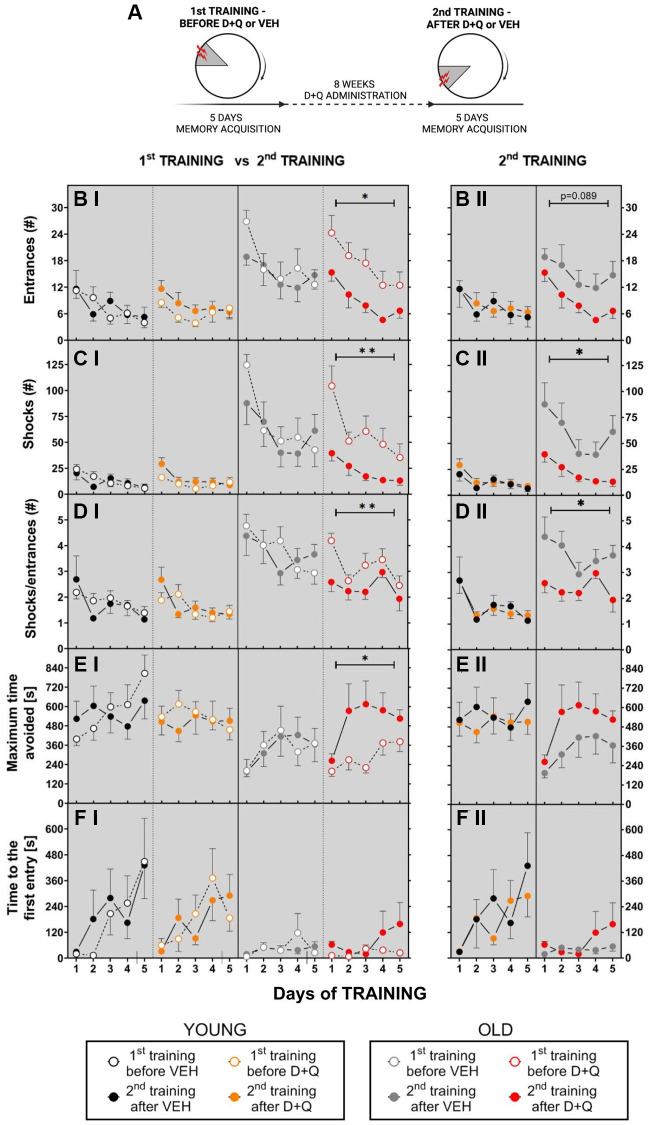
**Treatment with D+Q improved cognitive ability in aged rats tested in the AAPAT on spatial memory.** (**A**) Schematic diagram of the experimental design. Experiments consisted of 2 trainings, each with a different position of to-be-avoided place. The number of entries into the shock place (# of entries, **B**), the number of shocks (# shocks, **C**) and the number of shocks per one entry ratio (Shocks/entrance, **D**), together with maximum time avoided (**E**) and time to the first entry (**F**) during place avoidance training reflect changes in the learning abilities and memory in aged rats. Performance of place avoidance by young (3-mo old; YOUNG; *N* = 7) (black - vehicle, orange - D+Q) and aged (22-mo old; OLD; *N* = 7) (grey - vehicle, red - D+Q) rats in the AAPAT before and after 8 weeks of treatment with D+Q or vehicle (VEH) by oral gavage. Figures labeled with “**I**” depict comparison between 1st TRAINING and 2nd TRAINING, “**II**” between VEH and D+Q groups results of the 2nd TRAINING. The results are expressed as mean ± SEM, *N* = 7, ^*^p < 0.05, ^**^p < 0.01, ^***^p < 0.001. Data obtained for groups at consecutive trials were compared using repeated measures two-way ANOVA (2 groups and 5 days).

To study the effect of D+Q on aging-associated cognitive impairment, we designed an experiment in which allothetic spatial memory was tested in young and aged male Wistar rats (GROUP 1) in the active allothetic place avoidance task (AAPAT). The AAPAT was used to assess the acquisition of allothetic spatial memory before (1st TRAINING) and after the treatment with D+Q or vehicle (2nd TRAINING). The experimental outline is depicted in Figure 1A. It consisted of two place avoidance trainings, 5 training days each spaced by 8 weeks of vehicle or D+Q treatment period. It is important to notice that the position of the shock place during the 2nd TRAINING was described by new room frame coordinates in comparison to the 1st TRAINING. It therefore required the formation of a new spatial memory.

#### Aged rats presented worse memory acquisition in the place avoidance than young rats during the 1st TRAINING

We observed that aged non-treated rats assigned to vehicle or D+Q treatment groups (during 1st TRAINING - before treatment), learned the place avoidance in the AAPAT worse than young non-treated rats assigned to the respective vehicle or D+Q treatment groups and exhibited short-term memory impairment. The aged animals assigned to vehicle or D+Q treatment groups (during 1st TRAINING - before treatment), entered more often into the to-be-avoided place (made errors) and received more shocks than young rats assigned to the respective vehicle or D+Q treatment groups. The two-way ANOVA (4 groups and 5 days) for number of entrances and shocks confirmed main effect of groups (entrances F_(3, 26)_ = 9.718; *p* = 0.002; shocks F_(3, 25)_ = 21.60; *p* < 0.001) (Figure 1B I, 1C I). The value of each parameter depended on the day of the training and significantly changed during the training, which was confirmed by the two-way ANOVA for days. A detailed description of results for days is presented in the Supplementary Data (Supplementary Data 1).

Performance of place avoidance engages executive functions described by skill learning which was measured by shocks to entrances ratio (SHs/ENTRs). Low value of the ratio indicates effective skill learning. We found that aged rats presented worse skill learning than young rats. The two-way ANOVA (4 groups and 5 days) for SHs/ENTRs ratio confirmed main groups (F_(3, 26)_ = 34.61; *p* < 0.001) and days effect (F_(4, 104)_ = 6.65; *p* < 0.001) (Figure 1D I).

Aged rats presented worse short-term memory than young rats. It was manifested as shorter maximum time avoided between two entries into the shock place in aged rats. The same ANOVA for maximum time avoided confirmed the main effect of groups (F_(3, 26)_ = 4.19; *p* = 0.015) and days of training (F_(4, 104)_ = 5.57; *p* < 0.001) (Figure 1E).

Similarly, time to the first entrance (T1) differed between young and aged rats assigned to their respective vehicle or D+Q treatment groups. The two-way ANOVA (4 groups and 5 days) confirmed main groups (F_(3, 22)_ = 3.471; *p* = 0.033) and days effect (F_(4, 88)_ = 3.59; *p* = 0.009) (Figure 1E). Tukey’s post hoc multiple comparisons test for groups confirmed that both vehicle- and D+Q- treated young rats presented a longer T1 than aged rats assigned to vehicle and D+Q treatment groups (*p* = 0.056).

No differences were found between young rats assigned to the vehicle treatment group and young rats assigned to the D+Q treatment groups (the same applies to aged rats). On D1 length of time to the first entry was shorter than on D4 (Tukey’s multiple comparisons test; *p* = 0.02) (Figure 1).

#### Aged rats improved place avoidance acquisition and short–term memory after administration of D+Q

After 8 weeks of D+Q or vehicle administration the AAPAT was repeated with the new location of the shock place. Change of the shock place demanded formation of a new spatial representation of a to-be-avoided place (2nd TRAINING) (Figure 1A). D+Q treatment improved cognitive ability only in aged rats. Aged rats in the D+Q group exhibited significant place avoidance performance and short-term memory improvement relative not only to the vehicle group but also to their own results from pretreatment training (1st TRAINING).

The two-way ANOVA (2 groups and 5 days; aged vehicle 2nd TRAINING, aged D+Q 2nd TRAINING vs. 5 days) confirmed main effect of group for shocks (F_(1, 11)_ = 7.02; *p* = 0.022) in the 2nd TRAINING for aged vehicle and aged D+Q groups of rats. Aged D+Q rats received less shocks than aged VEH treatment rats (Figure 1C II). Moreover, aged D+Q treated rats presented effective skill learning (SHs/ENTRs) in comparison to aged VEH treated rats (F_(1, 11)_ = 9.1; *p* = 0.012) independent on training days (days effect NS) (Figure 1B II–1D II). Aged rats from the vehicle and D+Q group presented similar maximum time avoided and time to the first entrance on 2nd training (Figure 1E II and 1F II) (Supplementary Data 2).

Performance of aged rats in AAPAT before D+Q treatment was significantly different from that after D+Q treatment. The two-way ANOVA (2 trainings 1st TRAINING, 2nd TRAINING and 5 days) for entrances, shocks, skill learning and maximum time avoided confirmed main effect of trainings (entrances F_(1, 11)_ = 9.87; *p* = 0.009; shocks F_(1, 11)_ = 12.66; *p* = 0.005; SHs/ENTRs ratio F_(1, 11)_ = 8,87; *p* = 0.013) and maximum time avoided F_(1, 11)_ = 6.15; *p* = 0.03). Aged rats during 2nd TRAINING after D+Q treatment made a smaller number of ENTR, received less shocks and presented a low value of SHs/ENTRs ratio and a longer maximum time avoided compared to 1st TRAINING (before D+Q treatment) (Figure 1B I–1E I). Moreover, old rats from the D+Q group during 2nd TRAINING compared to their own results from the 1st TRAINING showed significant day effects for all the above parameters (Supplementary Data 2).

D+Q treatment did not influence performance of young rats neither when compared to the young vehicle group nor to their own results from the 1st TRAINING. The two-way ANOVA (2 groups and 5 days) for the number of entrances, shocks, shocks/entrances ratio and length of the maximum time avoided for young rats from the vehicle and D+Q groups showed non-significant main effect of groups. Effect of days was significant (Supplementary Data 3). Young rats regardless of treatment, performed place avoidance on a similar level and improved it in consecutive days of training (Supplementary Data 4).

Moreover, long-term memory evaluated by the time to first entrance on the-to-be-avoided place in young rats from vehicle and D+Q group before treatment as well as between 1st TRAINING and 2nd TRAINING for young D+Q rats was on a similar level (the same ANOVA shows non-significant groups, trainings or days effect) (Figure 1F I, 1F II).

D+Q treatment had no significant effect on body mass or locomotor abilities in young rats regardless of treatment (Supplementary Figure 1).

### D+Q treatment decreases peripheral inflammation including SASP factors in aged rats

**Figure 2:**
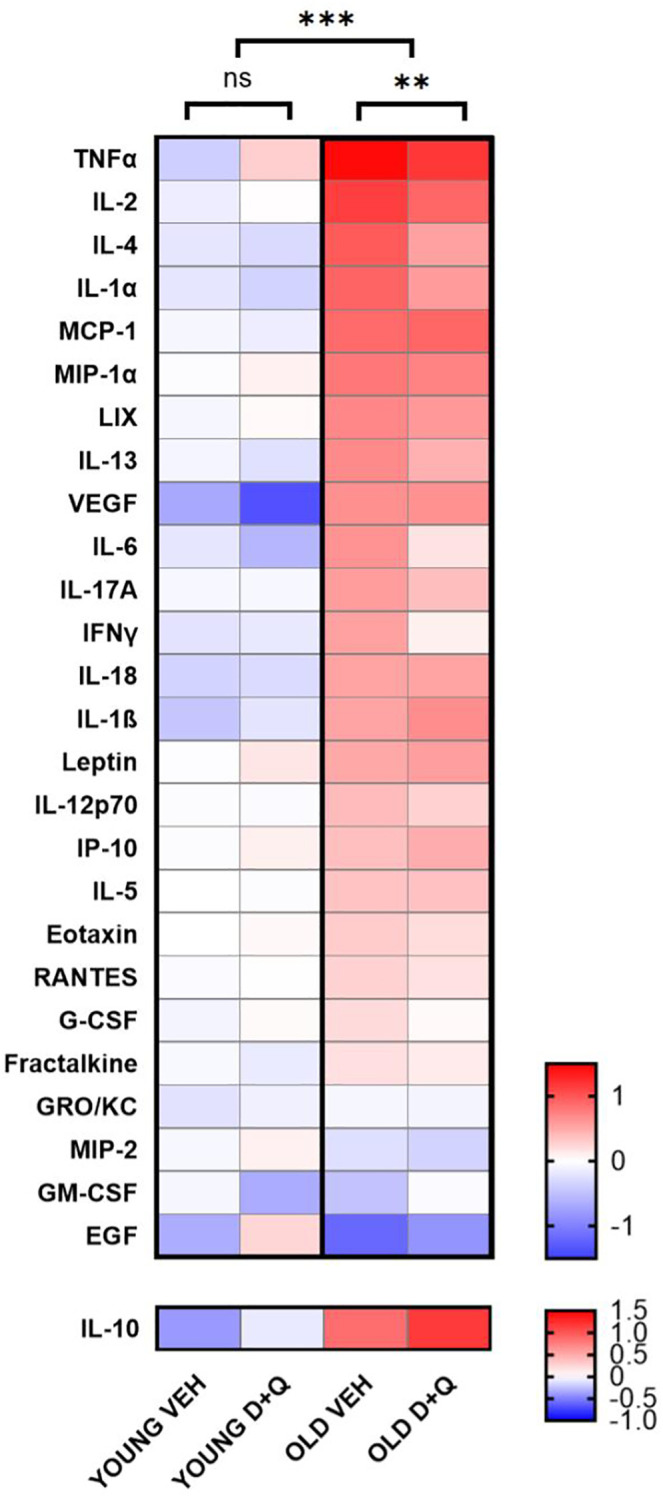
**D+Q treatment reduces peripheral inflammation in aged rats.** The heat map depicts cytokines and growth factor levels in blood serum collected after the final behavioral test from young and aged (6-month-old and 25-month-old respectively at the time of collection) rats treated with D+Q or vehicle (VEH). The results were normalized to the average of the young vehicle group. IL-10 as anti-inflammatory cytokine has been presented separately. The data were analyzed using two-way ANOVA; *n* = 7–8, ^*^p < 0.05, ^**^p < 0.01, ^***^p < 0.001.

Senolytics has been shown to reduce peripheral inflammation which is elevated in aged animals [27]. In order to confirm that D+Q treatment, in fact, alleviates low grade inflammation in rats we analyzed serum factors such as cytokine, chemokines and growth factors which belong to SASP [28]. As expected, aged rats showed generally higher inflammation, with analyzed serum factors being elevated compared to young rats (young vehicle vs. aged vehicle F_(1, 378)_ = 211.3; *p* < 0.001 and young D+Q vs. aged D+Q F_(1, 351)_ = 51.90; *p* < 0.001). Furthermore, D+Q treatment resulted in significant decrease in the levels of peripheral inflammatory mediators in aged (F_(1, 338)_ = 8.038; *p* = 0.005) but not young (F_(1, 364)_ = 0.4465; *p* = 0.504) rats. However, D+Q treatment did not result in a statistically significant change of any of the individual cytokines (Supplementary Figure 2). We did find significant differences in the levels of individual cytokines between young and aged rats, seven cytokines for vehicle treated groups (IL-1α, IL-β, IL-4, IL-2, IL-10, MCP-1 and TNF-α) and only two for D+Q treated groups (IL-10 and TNF-α) (Supplementary Figure 2). Interestingly IL-10, which is considered as the anti-inflammatory cytokine, was found to change on average in the opposite direction compared to other cytokines, further suggesting that D+Q treatment promotes an anti-inflammatory phenotype (Figure 2). In contrast to aged rats, serum level of cytokines and growth factors was not significantly affected by D+Q treatment in young rats.

### Improvement of cognitive skills in aged rats is associated with changes in synaptic plasticity in the stratum radiatum of CA1 region of the hippocampus

**Figure 3:**
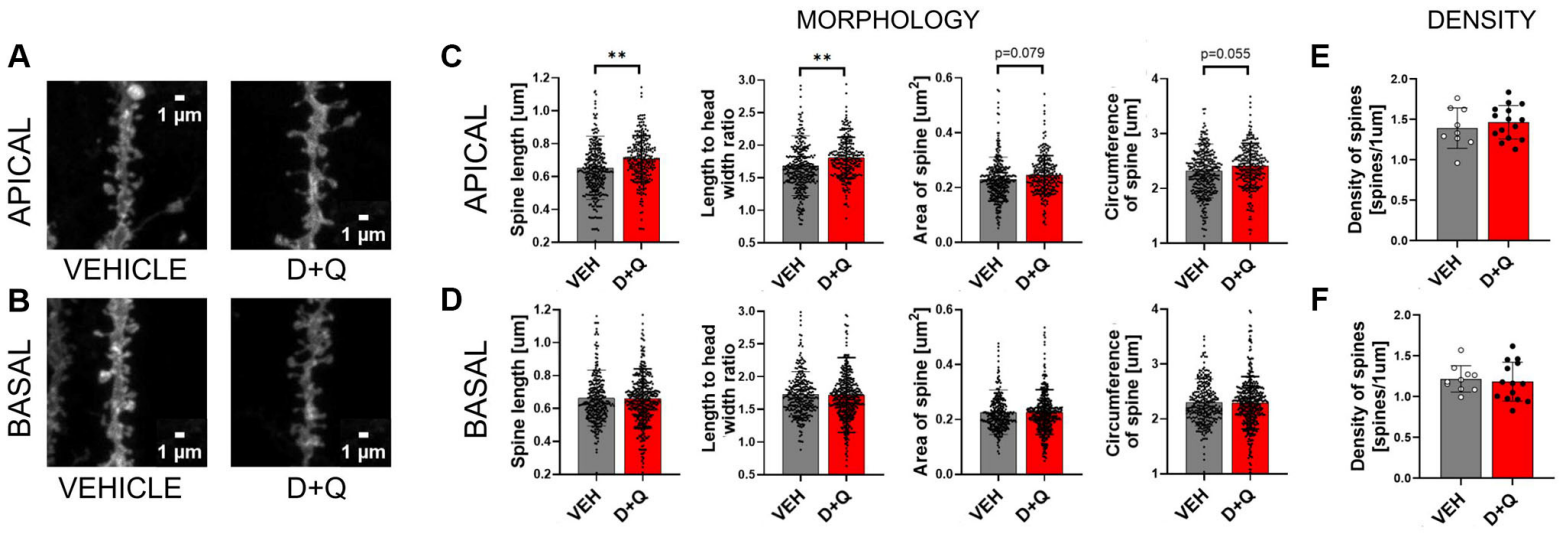
**D+Q treatment changes synaptic plasticity in the apical dendrites of neurons of the CA1 region of the hippocampus.** Representative images of DiI stained dendrites (**A** and **B**). DiI stained hippocampal slices from aged vehicle (grey bars) or D+Q (red bars) treated rats were used for the analysis of synaptic plasticity. Different parameters of the dendritic spine shape (length, length to width ratio, area, circumference **C** and **D**) and spine density (**E** and **F**) were analyzed in the CA1 region of the hippocampus. The analysis was done for two dendritic arbours of stratum pyramidale, namely basal (BASAL) (**D** and **F**) and apical (APICAL) dendrites (**C** and **E**). The data are expressed as mean ± SEM, and analyzed using nested *t*-test. Dots on the bar plots represent values for single dendritic spines (**C** and **D**) or single image (**E** and **F**) *N*_VEH_ = 3, *N*_D+Q_ = 5 animals, ^*^p < 0.05, ^**^p < 0.01, ^***^p < 0.001.

Synaptic plasticity is the activity-dependent modification of signal transmission between neurons. Changes in the synaptic transmission are associated with morphological alterations of the dendritic spines which constitute the postsynaptic part of the excitatory synapse. Synaptic plasticity, particularly in the hippocampal region, is critical for learning and memory formation. We hypothesized that improvement of cognitive abilities upon D+Q treatment should therefore be associated with changes in synaptic plasticity. To test this hypothesis, we analyzed dendritic spines morphology and density in DiI stained sections of hippocampus from aged vehicle and D+Q treated rats (Figure 3A and 3B). We found that dendritic spines on apical dendrites of the CA1 neurons underwent morphological changes after treatment with D+Q. The spines were longer (*p* = 0.008), and the ratio between the spine length and head width was significantly higher (*p* = 0.002) compared to the vehicle treated group. Moreover, we found a trend toward increase of the overall size of spines in the D+Q group as measured by the circumference and area of spines (circumference *p* = 0.055; area *p* = 0.079) (Figure 3C). Interestingly, these changes were specific to apical dendrites as we did not observe similar changes in basal dendrites of CA1 neurons between D+Q and vehicle groups of aged rats (length *p* = 0.209; length to width *p* = 0.453; circumference *p* = 0.364; area *p* = 0.186) (Figure 3D). Dendritic spines density was not affected by D+Q treatment neither in the stratum oriens (*p* = 0.692) nor in the stratum radiatum (*p* = 0.443) (Figure 3E and 3F).

### Beneficial effects of D+Q on cognitive skills in aged rats are accompanied by changes in histone H3 methylation in the hippocampus

**Figure 4:**
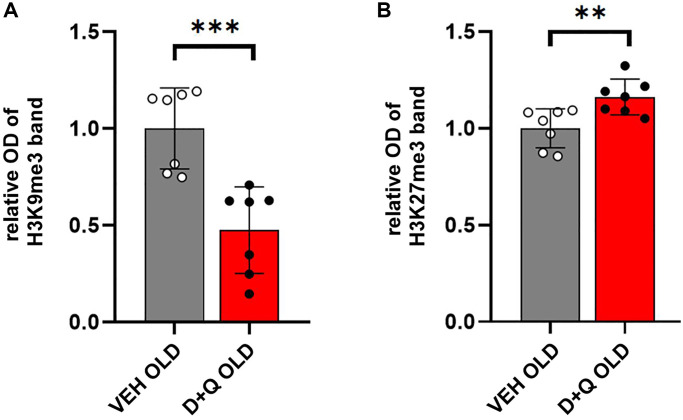
**Changes in the levels of H3 histones in the hippocampus of aged rats after D+Q treatment.** Improved cognitive skills, decreased peripheral inflammation, and changes in neuronal synaptic plasticity are accompanied by changes in H3 methylation profile. Trimethylation of H3K9 (**A**) and H3K27 (**B**) relative to the whole H3 in the hippocampus of aged rats after D+Q or VEH treatment measured by Western blot. The data are expressed as means ± SEM normalized to the average of the VEH group, and analyzed using *t*-test; *N* = 7, ^*^p < 0.05, ^**^p < 0.01, ^***^p < 0.001.

Altered epigenetic profile has been associated with age-dependent disorders. Methylation of histones H3 typically occurring at specific lysine (K) residues, such as H3K9, H3K27 regulates chromatin state and plays an important role in the regulation of gene expression. In particular, trimethylation of H3K9 (H3K9me3) and H3K27 (H3K27me3) which regulates silent heterochromatin stability has been implicated in age-dependent cognitive impairment [7, 8]. We hypothesized that rejuvenating effects of D+Q would affect the histone H3 methylation profile. To test this hypothesis, we performed Western blot analysis of histone extracts from hippocampal lysates of aged D+Q or vehicle treated rats. Our results showed a significant decrease in H3K9me3 levels relative to the total H3 in aged animals treated with D+Q (*p* < 0.001) (Figure 4A). Conversely, the levels of H3K27me3 were significantly higher in aged animals treated with D+Q compared to vehicle treated rats (*p* = 0.009) (Figure 4B).

### D+Q alleviates age-associated cognitive deficits in aged rats at least 5 weeks after discontinuation of the treatment

**Figure 5:**
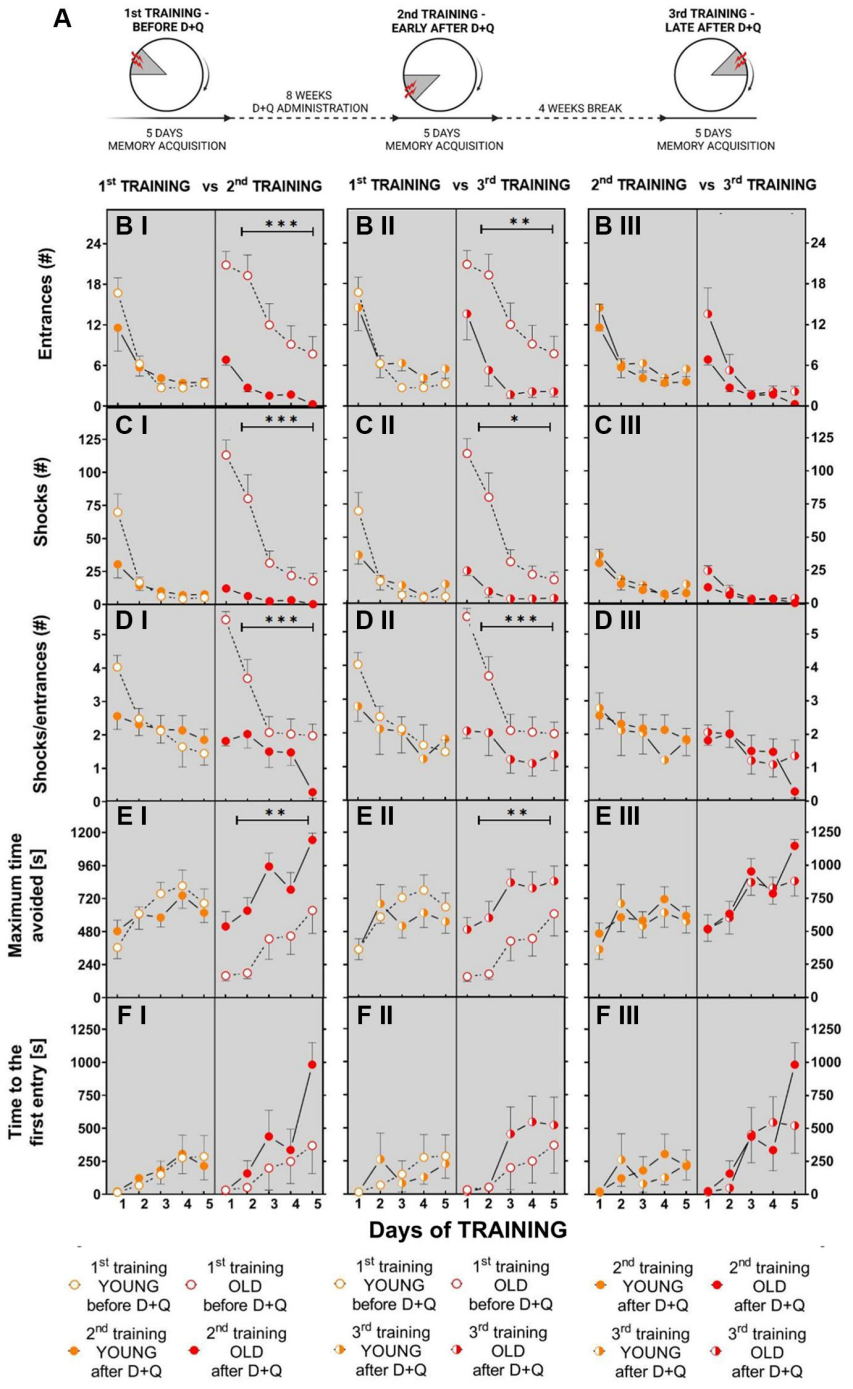
**Treatment with D+Q significantly improves cognitive abilities in aged rats in spatial memory task in the AAPAT on spatial memory for at least 5 weeks after treatment termination.** (**A**) Schematic diagram of the experimental design in which rats are subjected to the training 3 times, each time with a new position of the to-be-avoided place. The number of entries into the shock place (# of entries, **B I–II**I), the number of shocks (# shocks, **C I–III**) and number of shocks per one entry (Shocks/entrance, **D I–III**) together with maximum time avoided (**E I–III**) and time to the first entry (**F I–III**) during place avoidance training reflect changes in the learning abilities and memory in aged rats not only early after D+Q treatment (2nd TRAINING) but also after additional 4 weeks (3rd TRAINING). Performance of place avoidance by young (3-month old; YOUNG; orange color) and aged (18-month old; OLD; red color) rats in the AAPAT before and after 8 weeks of treatment with D+Q by oral gavage. Figures labeled with “**I**” depict comparison between 1st TRAINING and 2nd TRAINING, “**II**” between 1st TRAINING and 3rd TRAINING, whereas “**III**” depict comparison between 2nd TRAINING and 3rd TRAINING. The results are expressed as mean ± SEM, *N* = 7, ^*^p < 0.05, ^**^p < 0.01, ^***^p < 0.001. Data obtained for groups at consecutive trials were compared using repeated measures two-way ANOVA.

To test whether the beneficial effect of (D+Q) on cognitive functions is a long-lasting effect we performed another set of experiments in which aged and young Wistar rats (GROUP 2) were trained 3 times in the AAPAT, namely before D+Q treatment (1st TRAINING), early after D+Q administration (2nd TRAINING) and again 5 weeks (1 week of training + 4 weeks being left undisturbed in their home cage) after discontinuation of D+Q treatment (3rd TRAINING) (Figure 5A), each time to a new shock place.

Similarly to the first set of experiments, we found that aged rats learned the place avoidance task worse than young rats before treatment with D+Q during the 1st TRAINING. In the 1st TRAINING aged rats made more entrances into the shock place, with shorter maximum time avoided and received more shocks compared to young rats. The two-way ANOVA (2 groups and 5 days) for entrances, shocks and maximum time avoided on the 1st TRAINING confirmed main effect of groups (entrances F_(1, 11)_ = 6.11; *p* < 0.03; shocks F_(1, 12)_ = 11.62; *p* = 0.005; maximum time avoided F_(1, 12)_ = 6.41; *p* = 0.026). Performance of place avoidance changed across days (Supplementary Data 5).

The 2nd TRAINING of AAPAT with the new location of the to-be-avoided place started after 8 weeks of D+Q administration. During this training aged rats exhibited learning and memory improvements relative to their own results from the 1st TRAINING. (Figure 5B I–5E I). The two-way ANOVA (2 trainings 1st TRAINING, 2nd TRAINING and 5 days) for the number of entrances, shocks, skill learning and maximum time avoided confirmed main effect of the training (entrances F_(1, 12)_ = 19.71; *p* < 0.001; shocks F_(1, 12)_ = 28.54; *p* < 0,001; SHs/ENTRs ratio F_(1, 12)_ = 19.00; *p* < 0.001; maximum time avoided F_(1, 12)_ = 16.12; *p* = 0.002). The performance of AAPAT improved across days as shown by the significant days effect described with details in (Supplementary Data 6).

The same ANOVA for young rats has shown a lack of significant differences between 1st TRAINING and 2nd TRAINING for entrance, shocks, skill learning and maximum time avoided. Similarly to the aged rats, the young rats improved performance in AAPAT across days of training (Supplementary Data 7).

Results of the 3rd TRAINING revealed that aged rats retained improvements in cognitive abilities at nearly the same level as immediately after D+Q treatment (2nd TRAINING) (Figure 5B III–5E III). However, significant decrease in the number of entrances, number of shocks and increase of maximum time avoided were recorded during 3rd TRAINING in comparison to the 1st TRAINING in the group of aged rats (the same ANOVA (1st TRAINING, 3rd TRAINING *n* = 2 vs. days *n* = 5; entrances F_(1, 12)_ = 9.10; *p* = 0,01; shocks F_(1, 12)_ = 23.21; *p* < 0.001; SHs/ENTRs ratio F_(1, 12)_ = 15.13; *p* = 0.002; maximum time avoided F_(1, 12)_ = 9.96; *p* = 0.008) (Figure 5B II–5E II). During 3rd TRAINING rats received less shocks than on D1 and D2 of 1st TRAINING (Supplementary Data 8).

Due to the long duration of the experiments, a group of young rats reached approximately 7–8 months of age at the end of 3rd TRAINING when the final memory training to a new to-be-avoided place was performed. Treatment with D+Q and 3rd TRAINING performed after 5 weeks (1 week of training + 4 weeks being left undisturbed in their home cage) did not influence performance in the memory acquisition of AAPAT in the group of young rats to 2ndTRAINING (Figure 5B III–5F III). Rats presented similar performance of the avoidance on each day of 2nd and 3rd TRAINING, whereas the effect of days was significant for comparisons between 1st and 3rd TRAINING (Supplementary Data 9).

## DISCUSSION

The Geroscience Hypothesis asserts that the same molecular and cellular mechanisms are central to aging and age-related disorders [29]. Accumulation of senescent cells is considered as one of the key processes responsible for the functional deterioration in aging. A growing body of literature suggests that senolytic treatment may alleviate multiple geriatric syndromes and diseases occurring in advanced age [20]. Only recently the improvement of memory and learning skills upon clearance of senescent cells with the combination of D+Q in aged mice has been reported [25]. In the present study we show that 8 weeks of D+Q administration alleviated cognitive impairments associated with aging in Wistar rats. Aged rats following D+Q treatment displayed improved learning abilities and effective acquisition of spatial short- and long-term memory of a new place compared to vehicle-treated rats. Our results complement and extend previous reports on the beneficial effects of senolytics in treatment of age-related disorders. Previous data on D+Q action in aging has included mouse models (reviewed in [16], therefore to our knowledge, this is the first report describing the use of those drugs in rats, in such a context. Rats could be considered a very useful animal model for studying aging-related brain conditions as they exhibit a significant age-dependent decrease in cognitive abilities [30]. Moreover, significantly elevated inflammation baseline level has been observed in aged rats [31]. Most importantly, rats are considered to be particularly suitable for measuring cognitive abilities in spatial tasks due to their natural affinity to interactions with spatial and social environments [32]. All the above arguments prompted us to use rats for the present research. We used outbred stock Wistar rats because, in our opinion, this animal model better represents the heterogeneity among older adults in the rate of decline of perceptual reasoning and processing speed. Aging is associated with significant heterogeneity in cognitive and noncognitive behavioral abilities among both laboratory animals and humans [33, 34]. By testing the same cohort of aged animals before and after D+Q treatment in the same spatial task but to the place described by the new spatial coordinates we could limit the effect of initial heterogeneity in our data. In the active allothetic place avoidance task rats learn to avoid the virtual place of the rotating arena where short-lasting, weak, sporadic electrical shocks are administered. This place is in a fixed position to the distal room cues, whereas the proximal cues from the arena and from the movement are misleading what requires stimuli segregation and engaging cognitive coordination processes [26, 35]. This makes the test more challenging compared to the commonly used test for studying spatial memory, such as the Morris water maze test, where proximal, misleading stimuli, e.g. defecation, urination, which could confuse spatial orientation are dissolved in water. It has been shown previously, by testing young-adult rats using the AAPAT, that spatial memory, which demands cognitive coordination, was very sensitive to different treatments, e.g. tetrodotoxin (TTX) unilateral hippocampal reversible blockade [36], application of NMDA antagonists such as memantine or MK-801 that cause memory dysfunctions [37], or silver nanoparticles [38]. We found AAPAT to be a complex and sensitive method to measure cognitive skills, particularly in aged rats, since it can be completed despite age-associated with a decrease in physical fitness, which could be a confounding factor in tests requiring physical effort to complete such as Morris water maze test.

One of the primary modes of action of senolytics is the clearance of senescent cells, which release mediators of inflammation such as cytokines, chemokines and growth factors. The decreasing burden of senescent cells has been shown to reduce peripheral inflammation [27]. Indeed, similarly to previously published results from mouse models, we show that aged but not young rats respond to senolytic treatment with a significant drop in the levels of serum factors associated with SASP. Even though removal of senescent cells from different organs and tissues by D+Q has been a well-described phenomenon there is relatively small literature on that subject for the central nervous system in normal aging. Among those reports, some data provide insight into the senolytic activity of D+Q in the brain. Tau-containing neurofibrillary tangles (NFT) that accumulate in the brains of Alzheimer’s Disease (AD) patients and mice models of neurodegeneration display cellular senescence-like transcriptomic profiles. Magnetic Resonance Imaging (MRI) and histopathological analyses of the brains from D+Q treated rTg(tauP301L)4510 transgenic mice showed reduction in the total Tau-containing neurofibrillary tangles (NFT) density what was associated with increased levels of synaptophysin and PSD 95 proteins expression [39]. Bussian et al. found that p16-positive astrocytes and microglia accumulate in the brains of mice model of tau-dependent neurodegenerative disease. By genetic or pharmacological clearance of these cells they managed to prevent gliosis, NFT deposition, degeneration of neurons and cognitive function deterioration [40]. In another study, in the brains of patients with AD and AD mouse models, Aβ plaque-associated oligodendrocyte progenitor cells but not astrocytes, microglia, or oligodendrocytes, exhibited a senescence-like phenotype characterized by the upregulation of p21, p16 protein expression, and senescence-associated-beta-galactosidase (SA-β-gal) activity. Senolytic treatment of AD mice selectively removed senescent cells from the plaque environment, reduced neuroinflammation, decreased Aβ load, and ameliorated cognitive deficits [23]. In an interesting paper by Ogrodnik et al., alleviation of anxiety symptoms in the high-fat diet fed mice was achieved by intra gavage treatment with D+Q [41]. In the recent study Ogrodnik et al. revealed age-associated increase in the percentage of p16-positive microglia and oligodendrocyte progenitor cells in the hippocampi of aged mice. Although whole body genetic elimination of p16-positive senescent cells resulted in similar improvement of learning and memory measured in stone T maze compared to D+Q treatment, the reduction of p16 was not observed in the microglial population of the hippocampus in D + Q-treated mice [25]. Those results suggest that the beneficial effects of D+Q treatment might be mediated by the decrease of senescence burden in the periphery rather than in the central nervous system. From the above cited studies emerges differential susceptibility of various brain regions and cell types to the treatment-dependent attenuation of senescence. This could partially explain why we were not able to detect the downregulation of cell senescence markers (Supplementary Figures 3 and 4). In depth analysis using more sensitive methods and better defined brain regions and cell types potentially could allow detection of the changes in the levels of the senescence markers upon D+Q treatment. Those changes could translate into behavioral effects since a relatively small number of senescent cells in a given tissue may profoundly contribute to the spreading of low grade inflammation [42] which in aged animals participate in the inflammaging [12]. Inflammaging, in turn, is connected with cognitive impairment, what was reported not only for animals, but also for humans [43]. Thus, reduction of the low grade inflammation state observed by us in the serum of D+Q treated animals may as well have both direct and indirect effects on the brain function via changes in the cardiovascular function [44]. Further studies are necessary to estimate the extent to which D+Q improves memory and learning skills by directly affecting the brain tissue in aged animals.

Storing and processing information in the neural network depends on the number and quality of functional connections in the brain. Hippocampus, a brain structure critical for learning and memory formation, is particularly susceptible to aging-associated disorders such as Alzheimer’s Disease [45]. Overall, the hippocampus retains its architecture during aging, however functional alterations, such as impairment of long-term potentiation (LTP) induction or maintenance are reflected in aberrant dendritic spine morphology and gene expression. Long and thin dendritic spines seem to be particularly susceptible to aging. Those spines create new synaptic connections making it possible for the neuronal network to encode new information [46]. We found that D+Q treatment shifts the spine morphology of CA1 hippocampal neurons towards less mature connections which may explain the improved ability of aged rats to learn how to avoid the shock place in the AAPAT. Interestingly, we found that these changes of dendritic spine morphology are exclusive to the apical dendrites and not basal dendrites. Basal and apical dendrites of the CA1hippocampal region, despite being branches of the same pyramidal cells, have distinct protein composition, biophysical properties and are characterized by different inputs [47]. However, to the best of our knowledge, little is known about the impact of aging on those two portions of the dendritic tree. One study revealed aberrant LTP in basal but not apical dendrites of hippocampal field CA1 in slices prepared from middle aged 7- to 10-month-old rats compared to 3-month-old young adults [48]. The relatively young age of animals and lack of morphological data makes it difficult to extrapolate it to the data presented here.

Dynamic plasticity of adult neurons requires specific, strictly regulated and rapid changes in the gene expression. One of the mechanisms that has been implicated in the regulation of expression of genes associated with synaptic plasticity is histone H3 methylation. In particular trimethylation of H3K27 and H3K9. Even though both are associated with transcription repression, multiple studies have shown age-associated downregulation of H3K27me3 and upregulation of H3K9me3 in brain tissue. Significant drop in H3K27me3 levels found in brains of 22-month-old BALB/c mice compared to 3-month-old animals is reversed by caloric restriction, a treatment that improves cognitive abilities in aged animals [8]. On the other hand, in young mice, elevated levels of H3K9me3 occurring in postoperative recovery after isoflurane anesthesia cause cognitive impairments in fear conditioning paradigm [49]. Accordingly, downregulation of H3K9me3 in the hippocampus by inhibition of SUV39H1 leads to memory improvements in aged mice. Interestingly, this downregulation of H3K9me3 is accompanied by an increase in both thin and stubby spine in the CA1 region only at apical and not at the basal dendritic tree, which stands in agreement with our findings [9]. Strikingly similar results concerning changes in histone methylation profile have been obtained in the acute and subchronic stress rat models. Rats restrained once or for 7 days (30 min/day) displayed reduced levels of H3K27me3 and increased basal levels of H3K9me3 in the hippocampus [50]. Since a plethora of published data have linked stress with aberrant synaptic plasticity, it is plausible that those two repressive marks play an important role in the regulation of neuronal connectivity [51]. One potential mechanism linking brain histone H3 trimethylation and synaptic plasticity is BDNF, a potent regulator of synaptic plasticity and neuronal activity. Changes in the BDNF expression have been shown to inversely correspond to increased H3K9me3 in a postoperative model of cognitive impairment [49] or downregulation in mice with inhibited activity of SUV39H1 [9]. Moreover, gavage administration of quercetin at doses of 20 and 50 mg/kg has been shown to result in a significant increase in the hippocampal BDNF mRNA [52]. Further studies are necessary to fully understand the role of histone methylation in the beneficial effects of D+Q on cognitive impairment in aged animals.

In summary, we found that D+Q treatment alleviates cognitive behavioral deficits in aged but not young Wistar male rats in the AAPAT, which is accompanied by decreased peripheral inflammation measured by serum concentration of cytokines and growth factors (including SASP factors). Despite the lack of evidence of decreased senescent cell burden in the brain tissue, we found that improvement in learning and memory coincides with changes at the synaptic plasticity level. Dendritic spines on the apical dendrites of CA1 neurons of the hippocampus become longer and less mature, which we believe translates into more efficient formation of new connections. Since connectivity between neurons is tightly regulated at the molecular level, any changes in that process require alterations in the gene expression. Indeed we found that, upon D+Q treatment, methylation of histone H3, at the lysines residues 9 and 27, which has been shown to play an important role in age-associated cognitive decline, is modified in aged rats compared to vehicle treated groups and it is similar to that seen in young animals. Our findings provide mechanistic insight into the beneficial effects of D+Q on cognitive abilities in advanced age that may contribute to future studies associated with treatment of aging-associated diseases.

## MATERIALS AND METHODS

### Animals

Male Wistar 3-month-old rats (*n* = 23), 18-month-old rats (*n* = 7) and 22-month-old (*n* = 16) were obtained from the animal house of the Mossakowski Medical Research Center, Polish Academy of Sciences (Warsaw, Poland) or from Janvier Labs (Le Genest-Saint Isle, France). Animals were housed 3–4 per cage under a 12 h/12 h light/dark cycle (lights on at 8:00 AM) with food and water available ad libitum. In the present study we used only male animals due to ready availability of aged rats of that sex.

### Experiment design

The experiment consisted of analyzing behavior of animals in the Active Allothetic Place Avoidance Task (AAPAT). The experiments were performed on two groups of animals. The first group (GROUP 1) consisted of *n* = 16 young (3-month-old rats at the beginning of the experiment) and *n* = 16 aged (22-month-old rats at the beginning of the experiment). Young and aged animals were divided into groups of 8 rats which were later assigned to D+Q or vehicle treatment. Animals were tested 2 times in the AAPAT: before D+Q or vehicle treatment (1st TRAINING) and directly after cessation of D+Q treatment (2nd TRAINING). 87% (7 out of 8) animals in each treatment cohort survived until the end of the experiment. The second group (GROUP 2) used in order to check the long-term effects of D+Q on learning and memory formation consisted of *n* = 7 aged (18-month-old rats at the beginning of the experiment) and *n* = 7 young (3-month-old rats at the beginning of the experiment) which were tested 3 times in the AAPAT: before D+Q or vehicle treatment (1st TRAINING), directly after cessation of D+Q treatment (2nd TRAINING) and 5 weeks after the treatment discontinuation (4 weeks past 2nd TRAINING end - 3rd TRAINING). 100% (7 out of 7) animals in each treatment cohort survived until the end of the experiment. Due to injuries, results from one aged rat from GROUP 1 (D+Q treatment cohort, 2nd training) and results of one young rat from GROUP 2 (D+Q treatment cohort, 3rd training) were either not recorded or excluded from the final analysis after confirming impaired basic locomotion in the open field test. The schematic representation of experimental designs for each group are depicted in Figure 1A and Figure 5A, respectively. Before spatial memory acquisition training, all animals were habituated to the arena by placing them on the still arena without shock delivery for 10 min daily. After 5 days of habituation, spatial memory acquisition training in AAPAT began, with one 20 min session daily for 5 days. During the 1st TRAINING shocks were delivered within a virtual sector of the arena according to room-frame coordinates (northwest). After the 1st TRAINING, the rats received 5 mg/kg dasatinib (LC Laboratories) plus 50 mg/kg quercetin (Sigma) dissolved in 60% Phosal, 10% ethanol, and 30% PEG-400 or solvent (vehicle) alone (60% Phosal, 10% ethanol, and 30% PEG-400) in equivalent volume. The D+Q solution or vehicle was administered by oral gavage for 5 days per week every 8 weeks. After D+Q treatment, 2nd TRAINING of active place avoidance began, with a new position of the shock place (i.e., southwest). After the 2nd TRAINING, GROUP 2 was left undisturbed for another 4 weeks. After this 4-week period, AAPAT was conducted again (3rd TRAINING), this time with a shock place at the new position (i.e., northeast). The body mass of the animals was monitored throughout the experiment. At the end of 2nd TRAINING in group first Open Field Test were performed. Animals were euthanized on the next day after the last behavioral test.

### Analysis of cytokines and growth factors in blood serum

Arterial blood was collected in tubes containing a clot activator from heart puncture at the end of the study. Within an hour, the blood was centrifuged, aliquoted and stored at −80°C. Serum was analyzed by Eve Technologies (Calgary, Canada) using the Multiplex assay (Millipore) in two technical replicates. The following cytokines and growth factors were analyzed: tumor necrosis factor alpha (TNFα), interleukin-1 alpha IL-1α), IL-1β, IL-2, IL-4, IL-5, IL-6, IL-18, IL-10, IL-12, IL-13, IL-17A, monocyte chemoattractant protein-1 (MCP-1), macrophage inflammatory protein 1-alpha (MIP-1α), MIP-2, regulated upon activation, normal T-cell expressed and secreted (RANTES), C-X-C motif chemokine 5 (LIX), fractalkine, interferon gamma-induced protein 10 (IP-10), leptin, eotaxin, interferon γ (IFNγ), granulocyte-colony stimulating factor (G-CSF), granulocyte-macrophage colony-stimulating factor GM-CSF, human growth-regulated oncogene/keratinocyte chemoattractant (GRO/KC), and vascular endothelial growth factor (VEGF). Out of range values were randomly sampled from the range between 0 and the lowest value of the standard curve used to calculate serum factors concentrations. For statistical analysis we used log2 of raw values normalized to the average of the young vehicle group. Bar plots for each of the analytes can be found in Supplementary Figure 2.

### DiI staining of brain slices and synaptic plasticity analysis

To visualize changes in the shape of dendritic spines, 1,1′-dioctadecyl-3,3,3,3′-tetramethylindocarbocyanine perchlorate (DiI) staining was performed on hippocampal sections. The rat brain tissue was fixed in 4% PFA. The brains were dissected and sliced using a vibratome. Slices (140 μm thick) were allowed to recover for at least 1.5 h at room temperature in PBS. Random dendrite labeling was performed using 1.6 μm tungsten particles (Bio-Rad, Hercules, CA, USA) that were coated with propelled lipophilic fluorescent dye (DiI; Invitrogen) and was delivered to the cells by gene gun (Bio-Rad) bombardment. Images of dendrites in the hippocampus were acquired under 561 nm fluorescent illumination using a confocal microscope (63× objective, 1.4 NA) at a pixel resolution of 1024 × 1024 with a 3.43 zoom, resulting in a 0.07 μm pixel size. The images that were acquired from the brain slices were processed using ImageJ software (National Institutes of Health, Bethesda, MD, USA) and analyzed semi-automatically using custom-written SpineMagick software (patent no. WO/2013/021001). The analyzed dendritic spines belonged to secondary and ternary dendrites to reduce possible differences in spine morphology that are caused by the location of spines on dendrites with different ranks. Moreover, spontaneous changes in dendritic spine shape can obscure systematic effects because of the spontaneous intrinsic fluctuation of dendritic spine shape. To minimize this effect in the analysis, we used a scale-free parameter of relative changes in the spine length-to-head width ratio, which reflects spine shape. The spine length was determined by measuring the curvilinear length along a fitted virtual skeleton of the spine. The fitting procedure was performed by looking for a curve along which integrated fluorescence was at a maximum. The head width was defined as the diameter of the largest spine section while excluding the bottom part of the spine (1/3 of the spine length adjacent to the dendrite). Dendritic segments of at least 3 animals per condition were morphologically analyzed resulting in 4400 (VEH) and 6200 (D+Q) spines. To determine spine density, approximately 900 (VEH) and 1500 (D+Q) μm of dendritic length was analyzed per experimental group.

### Histone isolation, Western blotting and quantification

Histones were isolated from frozen hippocampi. First, the tissue was homogenized in lysis buffer (250 mM sucrose, 50 mM TrisHCl, pH 7.5, 25 mM KCl with protease (Roche 04693159001) and phosphatase (Roche 04906837001) inhibitors (added immediately prior to use) using glass homogenizer (15–20 strokes). Homogenate was then centrifuged at 7000 g for 1 min and the pellet containing nuclear fraction was resuspended in an extraction buffer (0.5N HCl, 10% glycerol). After 1 h, samples were centrifuged at 12100 g for 5 min. Histones were precipitated from the supernatant with acetone and pelleted by centrifugation at 12100 g for 5 min. Dried pellet was resuspended in 50 mM TrisHCl with 3% SDS. Isolated protein concentration was determined by BCA assay. The isolated histone fractions were then denatured in a sample buffer heated to 95°C for 5 min. About 10 ug of histone fractions were separated by a 15% (w/v) SDS-PAGE and transferred onto a nitrocellulose membrane (Amersham GE Healthcare). After blocking the membrane with 5% (w/v) non-fat milk in Tris-Buffered Saline Tween-20 (TBST), the membrane was incubated overnight with primary antibody, washed in TBST, incubated with Horseradish peroxidase (HRP) conjugated secondary antibody (anti-mouse and anti-rabbit, Dako Denmark A/S) at 1:2000 dilution in milk, washed and developed with ECL (Thermo Fisher Scientific). To obtain total H3 the membranes were stripped with a fresh stripping buffer (1.5% glycine, 0.1% SDS, 1% Tween, HCl, pH 2.2) for 20 min, washed twice with PBS and twice with TBST. Then, the blotting was conducted as described above. The following primary antibodies were used: H3 (Abcam ab1791, 1:4000 dilution), H3K27me3 (Diagenode C15410195, 1:1000 dilution), H3K9me3 (Diagenode C15410193, 1:1000 dilution). Developed films were scanned and signal intensities were quantified using Image J (National Institutes of Health, Bethesda, MD, USA).

### Active allothetic place avoidance test (AAPAT) – detailed description of the method

The place avoidance apparatus was described in detail in previous publications [26, 36]. The apparatus consisted of an 80-cm-diameter, elevated (80 cm), rotating (1 rpm) metal arena rimmed by a metal lip on the periphery (2 cm). The arena was located in the center of a 3 m × 4 m room with dim light (±24 l×) and explicit visual landmarks. Infrared light-emitting diodes (LED) were attached to the latex harness on the rat’s back. A computer system tracked the light-emitting diode position in the reference frame of the room every 20 ms by using an infrared-sensitive TV camera. The room frame position of a second LED fixed to the periphery of the arena also was tracked and was used to calculate the rat’s position in the reference frame of the arena. Thus, a to-be-avoided sector was defined in both room and arena reference frame. Before the experiment, rats were implanted with a 25-gauge (0.50 mm) hypodermic needle, puncturing the skin fold on their backs. The sharp end of the needle was then cut off and a small loop was formed with tweezers. This loop was connected by a mini-alligator clip that was attached with a cable to a shock box that was used for the delivery of electric shocks. Every time the rat entered the to-be-avoided sector (60 degrees), the computer triggered a mild, constant current (50 Hz, 0.5 s) foot-shock that was delivered across the low- and high impedance electrodes. The low-impedance (~100 Ω) shock electrode was clipped to the needle loop on the back of the rat, while the high-impedance (~100 KΩ) electrode was produced by contact of the rat’s feet to the grounded arena surface. The intensity of current was individually adjusted for each animal to provoke an escape reaction, ranging between 0.2 and 0.3 mA (50 Hz). If the rat did not leave the sector, the shock was repeated every 1.5 s. Commercial software was used for data collection and analysis (BioSignal Group, USA).

The AAPAT is a variant of the original place avoidance test (Bures et al., 1997). In the place avoidance task, locating the shock sector requires place navigation ability, and the execution of avoidance requires an instrumental response. Compared with standard spatial memory tests, the AAPAT is unique because the formation of spatial representation of a to-be-avoided place on a rotating arena requires ongoing segregation of room relevant stimuli from misleading. Spatial memory in the AAPAT was evaluated based on such parameters as: number of entrances, number of shocks on the shocks place, maximum time avoided and time to the first entrance into the shock place. Reduction of the number of entries (errors) and a reduction of the number of short-lasting shocks that were repeated until the rat escaped the shock place described effective acquisition of spatial memory, whereas long maximum time avoided expressed functioning of short-term memory. The increase in the time to the first entry into the shock place reflected the formation of long-term memory traces on consecutive training days.

### Open-field test

All open-field testing took place inside a close, well lighted room with minimal sound noise. The open-field arena (100 cm (l) 100 cm (w) 40 cm (h)) consisted of four opaque walls and a black matte floor. Prior to testing each animal, the entire open-field arena was cleaned using 70% alcohol. All animals were tested in the same manner, in brief, every animal was carefully placed near the same corner facing the wall. Video recording started prior to depositing the animal in the arena while analysis of the video footage started 15 seconds after. This was done to allow the experimenter time to leave the room and the animal to resume its behavior following the closing of the door. Video footage was analyzed using Toxtrac (https://sourceforge.net/projects/toxtrac). Parameters include det.mins = 3000, det.maxs = 50000, det.thr1 = 150, det.thr2 = 240. For exploration rate, the exploration was set as 100 mm in all edge settings which allows for dividing the whole arena in 10 cm^2^ squares. Every time the animal entered one square it counted as one explored square.

### SA-β-galactosidase activity

PFA fixed brains of either vehicle or D+Q treated rats were cut into 30 μm slices. Hippocampi were isolated and SA-β-galactosidase activity was measured. Slices were fixed in a buffer containing unmoved formaldehyde and 8% glutaraldehyde in PBS for 10 minutes, rinsed three times in PBS and incubated with x-gal buffer at 37°C in an air-tight box for 8 h. After SA-β-galactosidase detection, slices were processed for immunofluorescence. Astrocytes and neurons were targeted by immunofluorescence prior to SA-β-galactosidase staining with either anti-GFAP or anti-NeuN antibodies to define regions of interest by extracting the sum projection of either staining after thresholding the signal intensity using Fiji (ImageJ) software (National Institutes of Health, Bethesda, MD, USA). Within each region of interest, mean grey value of SA-β-galactosidase intensity was measured using the same software.

### Statistical analysis

Two compare data pairs we used Mann–Whitney test as a nonparametric, Student’s *t* test for parametric data. Nested *t*-test was used for the analysis of spine morphology. For analysis of AAPAT 5 day training curves or serum cytokine profiles between VEH and D+Q within one age group we used two-way ANOVAs followed by Tukey post hoc test. For analysis of cytokine profiles between young and aged rats we used two-way ANOVAs followed by Tukey post hoc test. *P* values lower than 0.05 were considered significant. All statistical tests were performed using GraphPad Prism 8 (GraphPad, San Diego, CA, USA). Data are presented as mean +/− SEM.

### Data availability statement

Data available on request from the authors.

## Supplementary Materials

Supplementary Data

Supplementary Figures
