# Supplementary material for: Combination of dasatinib and quercetin improves cognitive abilities in aged male Wistar rats, alleviates inflammation and changes hippocampal synaptic plasticity and histone H3 methylation profile
Source: Aging (Albany NY). 2022 Jan 18;14(2):572–95. doi: 10.18632/aging.203835 (PMC8833137; doi:10.18632/aging.203835)
Supplement: Supplementary Data [file aging-14-203835-s001.pdf]

## SUPPLEMENTARY DATA

Aged rats presented worse memory acquisition in the place avoidance than young rats during the 1st TRAINING.

**Supplementary Data 1**

Aged non-treated rats assigned to vehicle or D+Q treatment groups (during 1st TRAINING - before treatment), learned the place avoidance in the AAPAT worse than young non-treated rats assigned to the respective vehicle or D+Q treatment groups and exhibited short-term memory impairment. However, all rats improved place avoidance across training days. The two-way ANOVA (groups $n = 4$ vs. days $n = 5$) for number of entrances and shocks confirmed effect of training days (entrances $F_{(4,\ 104)} = 12.09$; $p < 0.001$; shocks $F_{(4,\ 100)} = 23.19$; $p < 0.001$) (Figure 1B I, 1C I). The number of entrances and shocks decreased after D2 (Tukey's multiple comparisons test; $p < 0.001$). The post hoc Tukey's test for interaction groups vs. days ($F_{(12,\ 100)} = 3.23$; $p < 0.001$) for shocks confirmed that during the first training day all aged rats received more shocks than all young rats ($p < 0.001$), whereas on the last training day this difference was not significant (Figure 1B I, 1C I, 1E I).

Aged rats improved place avoidance acquisition and short–term memory after administration of D+Q.

**Supplementary Data 2A**

After 8 weeks of D+Q or vehicle administration the 2nd TRAINING of place avoidance was repeated with the new location of the shock place (2nd TRAINING) (Figure 1A). D+Q treatment improved cognitive ability only in aged rats. However, two way ANOVA (groups $n = 2$ vs. days $n = 5$; aged VEH 2nd TRAINING, aged D+Q 2nd TRAINING vs. 5 days) confirmed significant day effects for entrances ($F_{(4,\ 40)} = 5.07$; $p = 0.002$), shocks ($F_{(4,\ 44)} = 4.39$; $p = 0.004$) , maximum time avoided ($F_{(4,\ 43)} = 4{,}106$). Rats made more entrances on D1 than on D3, D4 and D5, received more shocks and presented shortened maximum time avoided on D1 than on D3 and D4 (Tukey's multiple comparisons test; $p <$ 0.01) (Figure 1B II, 1C II, 1E II).

**Supplementary Data 2B**

Old rats from the D+Q group during 2nd TRAINING compared to their own results from the 1st TRAINING showed significant day effect for entrances (ENTR $F_{(4,\ 43)} = 6.37$; $p < 0.01$), shocks ($F_{(4,\ 43)} = 5.86$; $p < 0.001$), shocks/entrances ratio ($F_{(4,\ 43)} = 1{,}09$; $p < 0.01$) and maximum time avoided ($F_{(4,\ 44)} = 3.73$; $p = 0.01$). Rats made more entrances and received more shocks on D1 than in the next training days (Tukey's multiple comparisons test; $p < 0.01$). Skill ratio was on a lower level on D5 than on D1 ($p < 0.009$).

**Supplementary Data 3**

D+Q treatment did not influence performance of young rats neither when compared to the young vehicle group nor to their own results from the 1st TRAINING. Although effect of groups was not significant but the two way ANOVA (groups $n = 2$ vs. days $n = 5$) showed a significant effect of days for entrances ($F_{(4,\ 56)} = 3.52$; $p < 0.012$), shocks ($F_{(4,\ 56)} = 6.34$; $p < 0{,}001$), SHs/ENTRs ratio ($F_{(4,\ 56)} = 4.82$; $p = 0.002$). The number of entrances on D4 and on D5 was lower than on D1 (Tukey's multiple comparisons test; $p < 0.02$), the number of shocks decreased after D1 ($p < 0.001$) and SHs/ENTRs ratio was lower on D4, D5 than on D1 ($p = 0.003$) (Figure 1B II, 1C II, 1D II).

**Supplementary Data 4**

Young rats before and after D+Q administration changed performance in consecutive days of training. The two way ANOVA (1st TRAINING, 2nd TRAINING vs. days $n = 5$) confirmed day effect for entrances $F_{(4,\ 56)} = 3.37$; $p = 0.015$; shocks $F_{(4,\ 56)} = 6.22$; $p < 0{,}001$; SHs/ENTRs $F_{(4,\ 56)} = 3.92$; $p = 0.007$). On D1 rats made more entrances, received more shocks and presented a high ratio than in the next training days (Tukey's multiple comparisons test; $p < 0.01$) (Figure 1B I, 1C I, 1D I).

D+Q alleviates age-associated cognitive deficits in aged rats at least 5 weeks after discontinuation of the treatment.

**Supplementary Data 5**

Similarly to the first set of experiments we found that aged rats learned the place avoidance task worse than young rats before treatment with D+Q during the 1st TRAINING. However, the two-way ANOVA (2 groups and 5 days) for entrances, shocks and maximum time avoided on the 1st TRAINING confirmed effect of days (entrances $F_{(4,\ 44)} = 41.70$; $p < 0.0001$; shocks $F_{(4,\ 48)} =$ 49, 29 $P < 0{,}0001$; maximum time avoided $F_{(4,\ 48)} =$ 9,293 $p < 0{,}0001$) (Figure 5B I, 5C I, 5D I).

All rats improved performance across days and presented less entrances, shocks and longer maximum time avoided on D3, D4 and on D5 compared to D1 and D2 (Tukey's test $p < 0.01$). Improvement of

performance over the course of days is evidence that tested rats retained the ability to learn and memorize despite their age. It was confirmed by non-significant group effect for skill learning evaluated as SHs/ENTRs ratio, whereas the day's effect was significant (two-way ANOVA $F_{(4, 48)} = 29.60$; $p < 0.001$). Rats improved skills from the third day of 1st TRAINING. Moreover, there was a significant groups vs. days interaction for entrances ($F_{(4, 44)} = 3.95$; $p = 0.008$) and shocks ($F_{(4, 48)} = 4.58$; $p = 0.003$). Aged rats on D2 and D3 made more entrances than young rats, whereas on D1 and D2 received more shocks than young rats at the same time points (the Tukey's multiple comparisons test; $p = 0.01$) (Figure 5B I, 5C I, 5D I).

**Supplementary Data 6**

The 2nd TRAINING of AAPAT with the new location of the to-be-avoided place started after 8 weeks of D+Q administration. During this training aged rats exhibited memory improvements relative to their own results from the 1st TRAINING (Figure 5B I, 5C I, 5D I, 5E I). Moreover the two-way ANOVA (2 trainings 1st TRAINING, 2nd TRAINING and 5 days) for the number of entrances, shocks, skill learning and maximum time avoided confirmed effect of days (entrances $F_{(4, 48)} = 40,06$; $p < 0,001$; shocks $F_{(4, 48)} = 38.14$; $p < 0.001$; SHs/ENTRs $F_{(4, 48)} = 19.09$; $p < 0.001$; maximum time avoided $F_{(4, 48)} = 13.37$; $p < 0.001$). The animals made fewer entrances with longer maximum time avoided on D4, D5 and received a lower number of shocks on D4 and on D5 than on D1, D2 and D3 (Tukey's multiple comparisons test; $p < 0.001$). Old D+Q treated rats presented improvement in skill learning on D1 and on D5 of 2nd TRAINING in comparison to the 1st TRAINING (post-hoc test $p < 0.001$ and $p < 0.001$ for SHs/ENTRs for interaction training vs. days $F_{(4, 48)} = 7.517$; $p < 0.001$). Rats entered the to-be-avoided place more often during the 1st TRAINING than during the 2nd TRAINING (post hoc $p < 0.001$ for interaction trainings vs. days ($F_{(4, 48)} = 9, 80$; $p < 0,001$). Numbers of shocks on D1 and on D2 were higher for 1st TRAINING than for the 2nd TRAINING (post hoc $p < 0.001$ for interaction trainings vs. days $F_{(4, 48)} = 25, 00$; $p < 0,001$).

**Supplementary Data 7**

The young rats have shown a lack of significant differences between 1st TRAINING and 2nd TRAINING for entrance, shocks, skill learning and maximum time avoided (Figure 5B I, 5C I, 5D I, 5E I). However, similarly to the aged rats, the young rats improved performance in AAPAT across days of training (entrances $F_{(4, 48)} = 20.41$; $p < 0.001$; shocks $F_{(4, 48)} = 20.38$; $p < 0.001$; SHs/ENTRs $F_{(4, 48)} = 6.09$; $p = 0.002$; maximum time avoided $F_{(4, 48)} = 4.99$; $p = 0.002$). The number of entrances decreased significantly from D2 to D5 in comparison to D1 (Tukey's multiple comparisons test; $p < 0.001$), whereas SHs/ENTRs ratio was low on D5 in comparison to D1 ($p = 0.006$). Tmax was long in the last three days of training.

Interaction training vs. days was significant for the number of shocks ($F_{(4, 48)} = 4, 96$; $p = 0.002$). The post hoc test confirmed that the young rats on D1 of the 1st TRAINING received a greater number of shocks than on D1 in the 2nd TRAINING furthermore, the number of shocks was decreasing in consecutive days of each training compared to D1 ($p < 0.001$).

**Supplementary Data 8**

Results of the 3rd TRAINING revealed that aged rats retained improvements in cognitive abilities at nearly the same level as immediately after D+Q treatment (2nd TRAINING). No significant differences were found between 2nd and 3rd TRAINING (Figure 5B III–5E III). However, a significant decrease in the number of entrance, the number of shocks, a low level of SHs/ENTRs ratio and an increase of maximum time avoided were recorded during 3rd TRAINING in comparison to the 1st TRAINING. The two way ANOVA (2 trainings 1st TRAINING, 3rd TRAINING and 5 days) confirmed significant effect of days (entrances $F_{(4, 48)} = 27.68$; $p < 0.001$; shocks $F_{(4, 48)} = 42.50$; $p < 0.001$; SHs/ENTRs ($F_{(4, 48)} = 12.47$; $p < 0.001$; maximum time avoided $F_{(4, 48)} = 8.14$; $p < 0.001$). Rats made less entrances, received less shocks and improved skills on D3, D4 and D5, compared to D1 and D2, whereas maximum time avoided was longer on D5 than on D1 and D2 (Tukey's multiple comparisons test; entrances $p = 0.01$; shocks $p = 0.01$; skills $p < 0.001$; maximum time avoided $p = 0.01$). Old rats on D1 and on D2 receive more shocks than during the next days of the 1st TRAINING and all days of the 3rd TRAINING (post hoc $p < 0.001$; for interaction training vs. days $F_{(4, 48)} = 18.84$; $p < 0.001$).

**Supplementary Data 9**

Due to the long duration of the experiments, a group of young rats reached approximately 7–8 months of age at the end of 3rd TRAINING when the final memory training to a new to-be-avoided place was performed. No differences in place avoidance was found in the group of young D+Q treated rats on 3rd TRAINING in comparison to 2nd TRAINING. However, the two way ANOVA (2 trainings 1st TRAINING, 3rd TRAINING and 5 days) for trainings confirmed significant effect of days for entrances ($F_{(4, 44)} = 23.70$; $p < 0.001$), shocks

($F_{(4, 44)} = 21.85$; $p < 0.001$) SHs/ENTRs $F_{(3, 206)} = 6.288$; $p = 0.001$; maximum time avoided $F_{(4, 44)} = 5.46$; $p = 0.001$). (Figure 5B II–5E II). On D1 rats made more entrances, received more shocks and presented higher SHs/ENTRs ratio than in the next few days (Tukey's multiple comparisons test; $p < 0,001$), whereas maximum time avoided significantly increased after D1 ($p < 0.001$).
