# Supplementary material for: Combination of dasatinib and quercetin improves cognitive abilities in aged male Wistar rats, alleviates inflammation and changes hippocampal synaptic plasticity and histone H3 methylation profile
Source: Aging (Albany NY). 2022 Jan 18;14(2):572–95. doi: 10.18632/aging.203835 (PMC8833137; doi:10.18632/aging.203835)
Supplement: Supplementary Figures [file aging-14-203835-s002.pdf]

SUPPLEMENTARY FIGURES

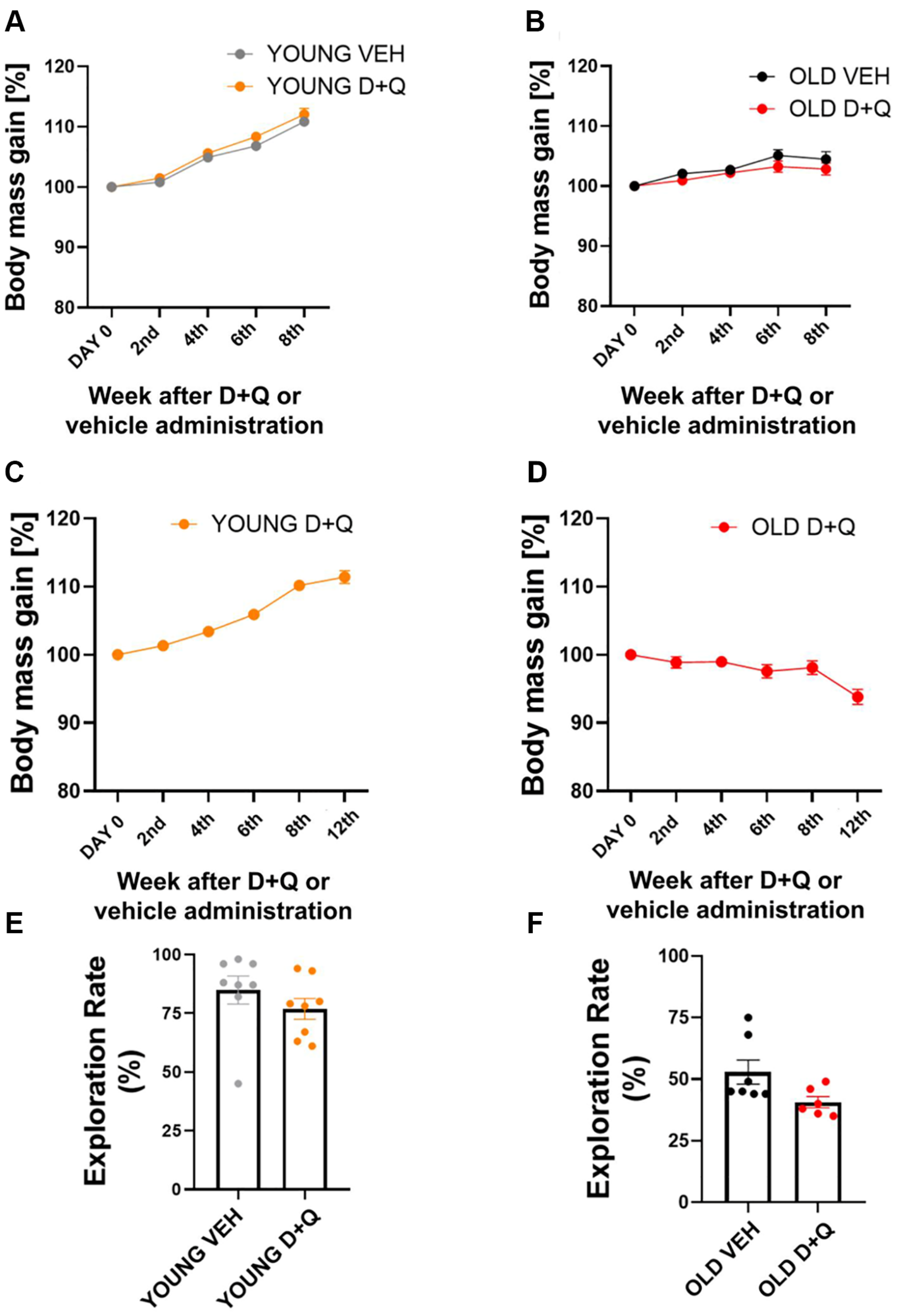

Supplementary Figure 1. D+Q treatment does not change long-term memory retrieval, locomotor activity in male Wistar rats. Relative body mass gain throughout the experiments was measured in relation to the body mass recorded immediately before administration of the first dose of D+Q or vehicle (VEH) (**A-D)**. Exploration rate in open field test was performed after the D+Q or vehicle treatment in young (**E**) and aged (**F**) animals. Exploration ratio was calculated by dividing the open field arena into 10 cm^2 squares and calculating the ratio of explored squares recorded with the Toxtrac software over the total number of squares for every individual of both mischiefs during the open field test. Data were analyzed by Two-way ANOVA followed by Tukey multiple comparison test for vehicle vs. D+Q groups before and after treatment with $^{*}p < 0.05$. The results are expressed as mean ± SEM, n N = 7–8.

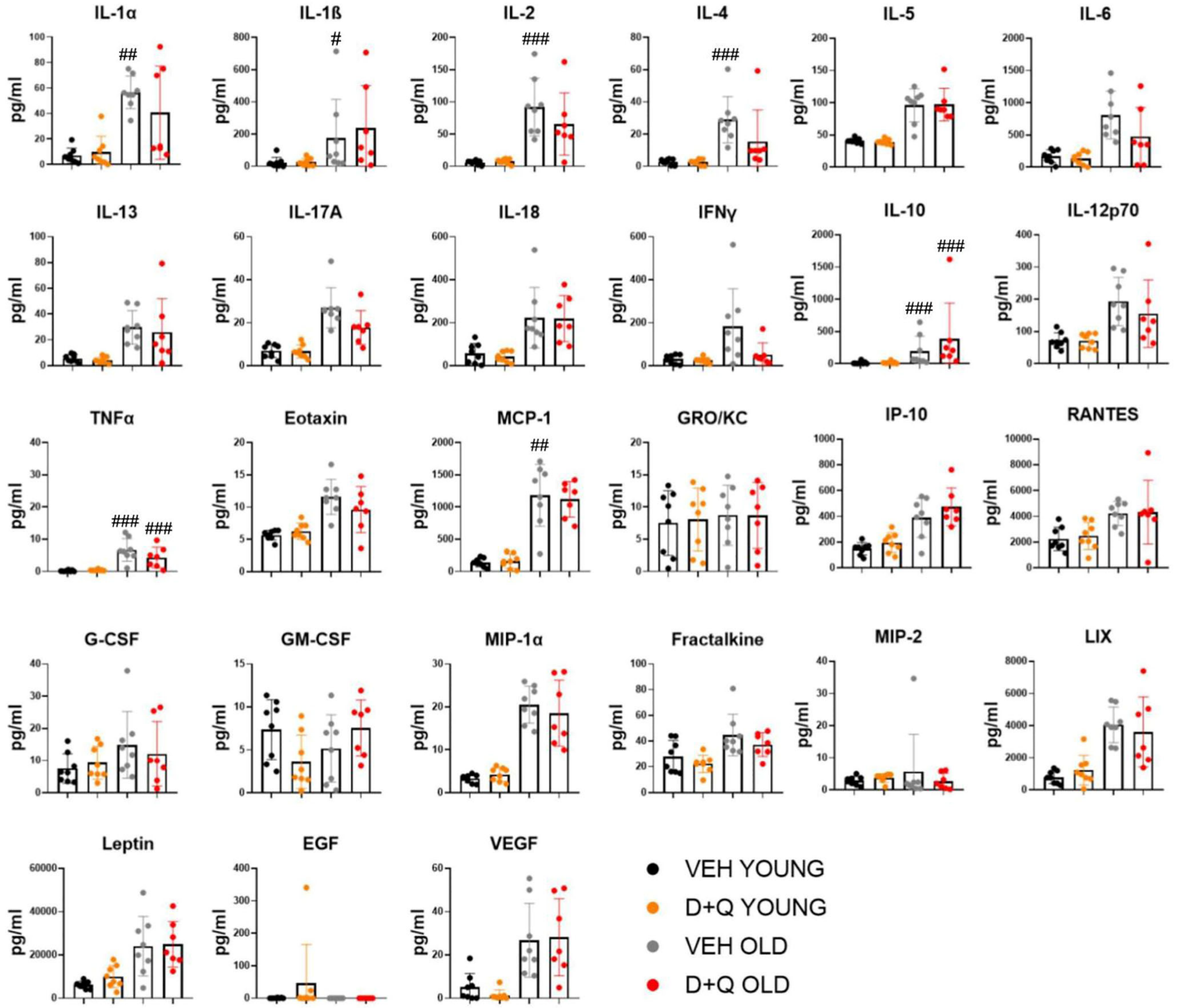

Supplementary Figure 2. Levels of each of the analytes measured in blood serum collected from young and aged animals after 8 week of D+Q or vehicle (VEH) administration. Each dot represents a mean value from two technical replicates. The results are expressed as mean ± SEM, *N* = 7–8. '#' indicate differences between young and aged groups where $^{\#}p < 0.05$, $^{\#\#}p < 0.01$, $^{\#\#\#}p < 0.001$.

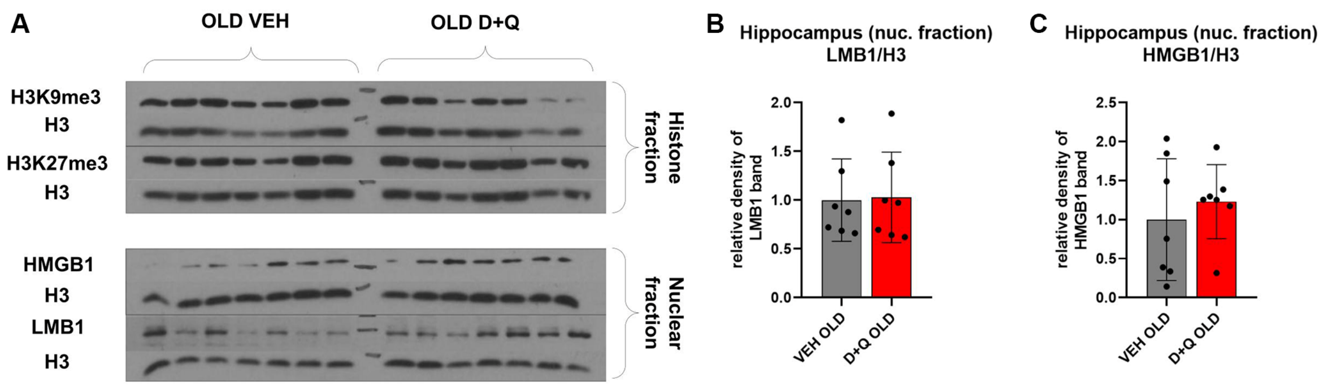

Supplementary Figure 3. Western blot results (**A**) Raw scans of Western blots of HMGB1, LMB1, H3K9me3 and H3K27me3 together with corresponding histone H3 level. Relative density of LMB1 (**B**) and HMGB1 (**C**) measured in the nuclear fraction of hippocampal homogenate from young and aged animals after 8 week of D+Q or vehicle (VEH) administration. Each dot represents the value obtained from one animal. The results are expressed as mean ± SEM. Results were normalized to the mean of the aged vehicle group (OLD VEH), $N = 7$.

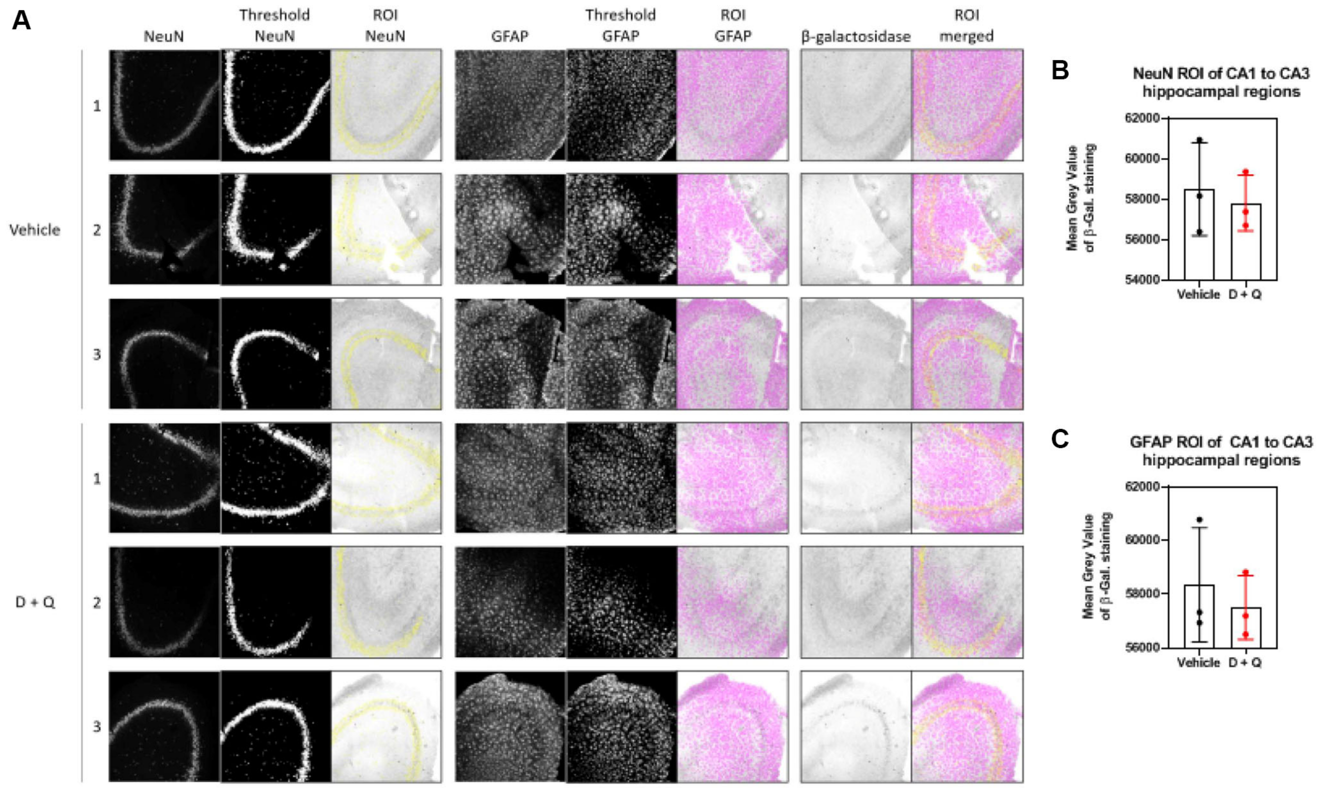

Supplementary Figure 4. SA-β-galactosidase expression in hippocampal CA1 to CA3 region of vehicle and D+Q treated rats. PFA fixed brain of vehicle and D+Q treated rats were cut in 30 µm slices. Hippocampi were isolated and SA-β-galactosidase staining was performed. Astrocytes and neurons were targeted by immunofluorescence prior to SA-β-galactosidase staining with either anti-GFAP or anti-NeuN antibodies to define regions of interest by thresholding the signal intensity using Fiji (ImageJ) software. Within each region of interest, mean grey value was measured using the same software. Representative images for each rat from both treatment groups showing sum projection of NeuN, GFAP and REST staining, thresholding of NeuN and GFAP along with the overlay of their individual and merged regions of interest (**A**). Mean grey value of SA-β-galactosidase staining in the NeuN region of interest from CA1 to CA3 hippocampal region (**B**). Mean grey value of SA-β-galactosidase staining in GFAP region of interest from CA1 to CA3 hippocampal region (**C**). Data were expressed as scattered dots bar-plots showing mean ± SEM. $N = 3$ rats per group comprising 2 images of individual hippocampal slices per animal across two separate immunofluorescence experiments. Data were determined by Student *t*-test between vehicle and D+Q.
